# Forensic view on two Raspberry Shake burglargrams

**DOI:** 10.1007/s10950-022-10098-5

**Published:** 2022-06-11

**Authors:** Klaus-G. Hinzen, Heinrich Krummel, Bernd Weber, Claus Fleischer

**Affiliations:** 1 Dep. Geosciences, University of Cologne, Cologne, Germany; 2 geoFact GmbH, Bonn, Germany; 3 Gempa GmbH, Potsdam, Germany

**Keywords:** Citizen seismological stations, Forensic seismology, Explosion, Airblast

## Abstract

A steadily increasing number of citizen seismological stations, often located in populated areas, record a plethora of man-made events. These events are especially of interest, when they are caused by criminal activity or man-made explosions. On 4 December 2021, during an attempted robbery of an automated teller machine (ATM) in Bonn-Röttgen, Germany, the burglars used explosions. The seismic effects of the explosions were recorded with a Raspberry Shake (RS) station at a distance of 580 m from the site. While working on the analysis of this signal, another attack on an ATM on 23 February 2022 in Kürten-Dürscheid was recorded by another RS station, this time at 830-m distance with an instrument that also included an air pressure channel. The seismic signatures of both events indicate similar procedures in both cases whereby a larger explosion was quickly followed a smaller explosion after 21 s and 49 s, respectively. An estimate of the charge weight of the explosions shows that ratios of the strength of the first to second explosion were 21:1 and 9.4:1 in the Röttgen and Dürscheid attacks, respectively.

## Introduction

**Fig. 1:**
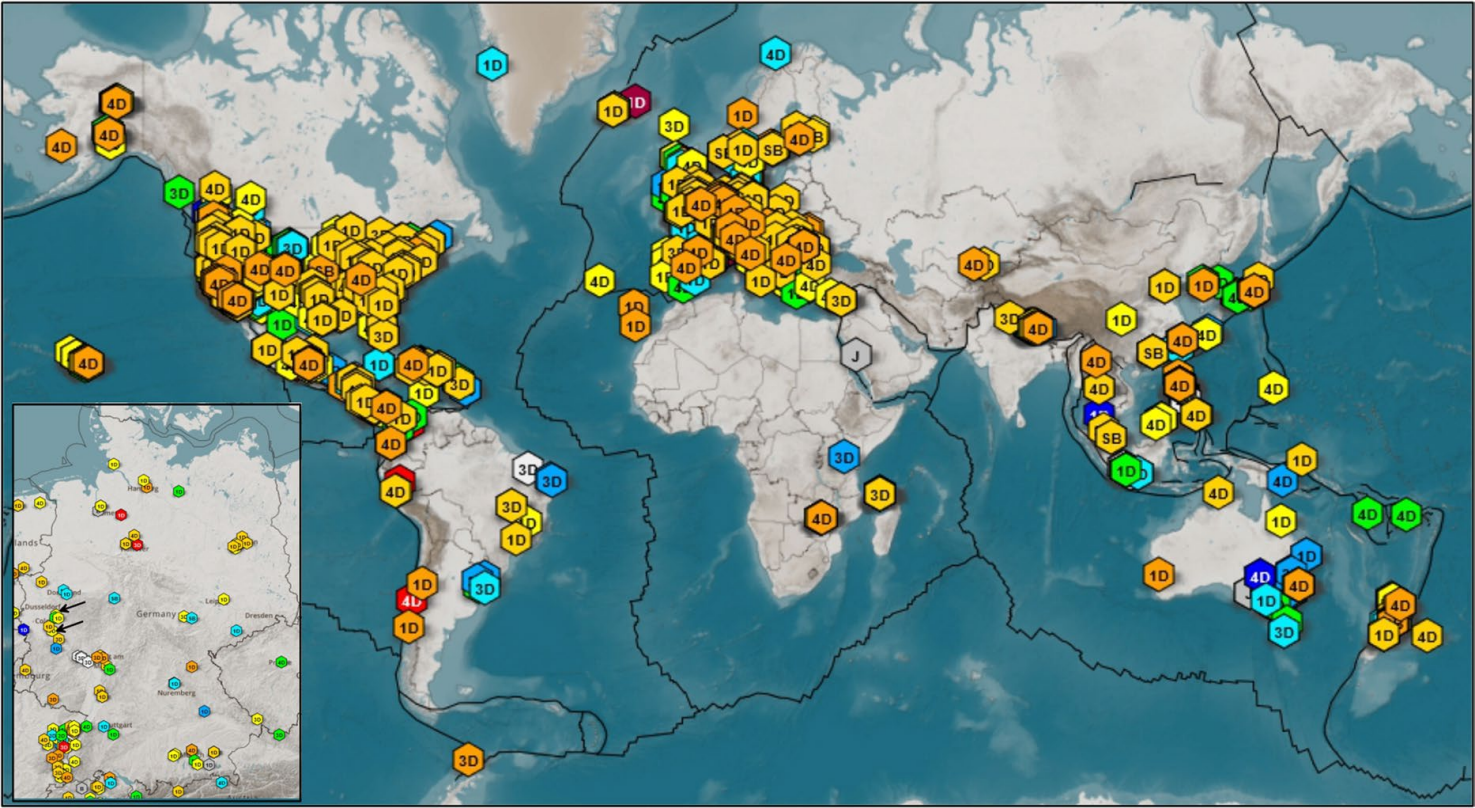
Raspberry Shake citizen stations active worldwide in December 2021. 1D, 3D, 4D, and SB indicate the sensor type. The insert on the lower left shows the RS stations in Germany, the stations used in this study are indicated by black arrows (https://community.raspberryshake.org/, last accessed 02.2022)

Citizen seismology has gained momentum in the past decade based on an increase in the number of available stations and by centralized and standardized data acquisition (e.g., Anthony et al. 2018; Chen et al. 2020). At the time of this writing, the Raspberry Shake (RS) community (https://community.raspberryshake.org/, last accessed 02.2022), for example, has more than 1622 participants (Fig. 1) and more than 60 RS stations are active in Germany (https://stationview.raspberryshake.org/, last accessed 01.2022). These stations include single (vertical) component, three component, and stations with an additional infrasound channel. While professional seismic stations are ideally located in remote places with low noise conditions, citizen stations tend to be located in populated areas, often in residential buildings. Thus, in addition to earthquake ground motions, local anthropogenic signals ranging from washing machines to regular bus and train traffic are recorded. One notable example is the study by Lecocq et al. (2020) who used citizen stations (among others) to illustrate the quieting effect of the COVID-19 pandemic on anthropogenic seismic noise.

The increasing density of citizen seismic stations provides a rich source of non-earthquake events for analysis, including that which forms the basis for forensic seismology (e.g., Thirlaway 1973; Douglas 2007). Initially the term “forensic seismology” was mainly associated with seismic research of nuclear tests for event discrimination and yield estimation (Bowers and Selby 2009). However, due to increased data availability, different types of seismic sources have been studied. These include recordings from spectacular events such as the Oklahoma City bombing (Holzer et al. 1996); the bomb attack on the US embassy in Nairobi, Kenya (Kopper et al. 1999); the sinking of the Russian submarine Kursk (Kopper et al. 2001); the attack on the World Trade Center (Kim et al. 2001); the Carlsbad, Texas pipeline explosion (Kopper et al. 2003); the London fuel tank explosion (Hinzen 2007); the explosion of the Chelyabinsk meteor (Heimann et al. 2013); the seismic signature of the deadly snow avalanche in Italy (Braun et al. 2020); or the explosion of ANFO (ammonium nitrate fuel oil) in Beirut, Lebanon (Ghalib et al. 2021). Smaller events have also been studied such as a lightning strike next to a seismic station (Hinzen 2012) or an accidental Second World War (WW2) bomb explosion in Euskirchen, Germany (Hinzen 2014). Crime scenes can also generate a seismic record. The term “burglargram” from the title of this paper was first used by Hinzen et al. (2015) who analyzed the seismic data recorded during a burglary at the seismological station BNS of University of Cologne. (The term was suggested during the review process by Susan Hough and immediately adapted by the authors.)

**Fig. 2:**
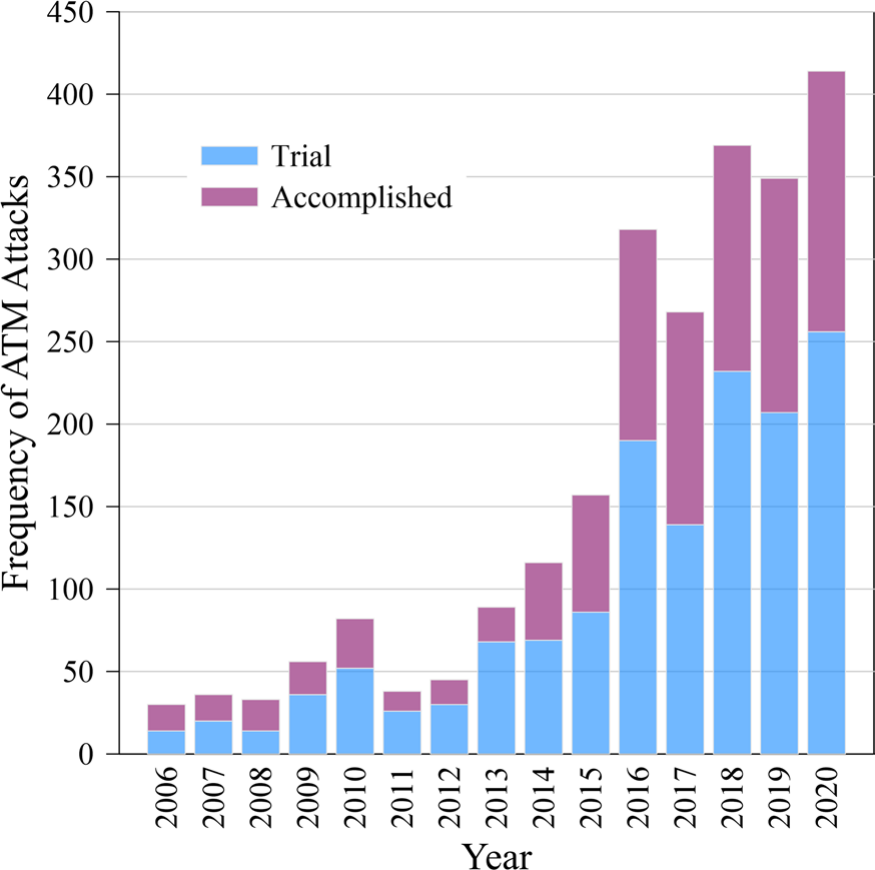
Frequency of ATM attacks in Germany with the use of explosions from year 2006 to 2020. Source: see “Data availability”

In Germany, the number of robbery attempts on automatic teller machines (ATMs) has increased dramatically over the past decade (Fig. 2). While 30 such attacks per year occurred from 2006 to 2008, in 2020 the Federal Criminal Police Office reported 704 attacks, with explosives involved in 414 cases. Of those, 256 attacks resulted in completed thefts and 158 failed. A commonly used robbery technique is filling the machines with a gas and igniting it to cause an explosion. However, banks have responded with countermeasures such as gas neutralizing devices and a color marking system that renders the cash unusable. Unfortunately, the gas neutralization had the effect of increasing the use of solid explosives from 18 in 2019 to 111 in 2020 (https://www.bka.de/DE/Presse/Listenseite_Pressemitteilungen/2021/Presse2021/210615_pmBLBGeldautomaten.html, last accessed 01.2022). Solid explosives caused damages which greatly exceed the amount of stolen cash (17.1 Mio €) in addition to the risk that innocent pedestrians or residents are injured.

**Fig. 3:**
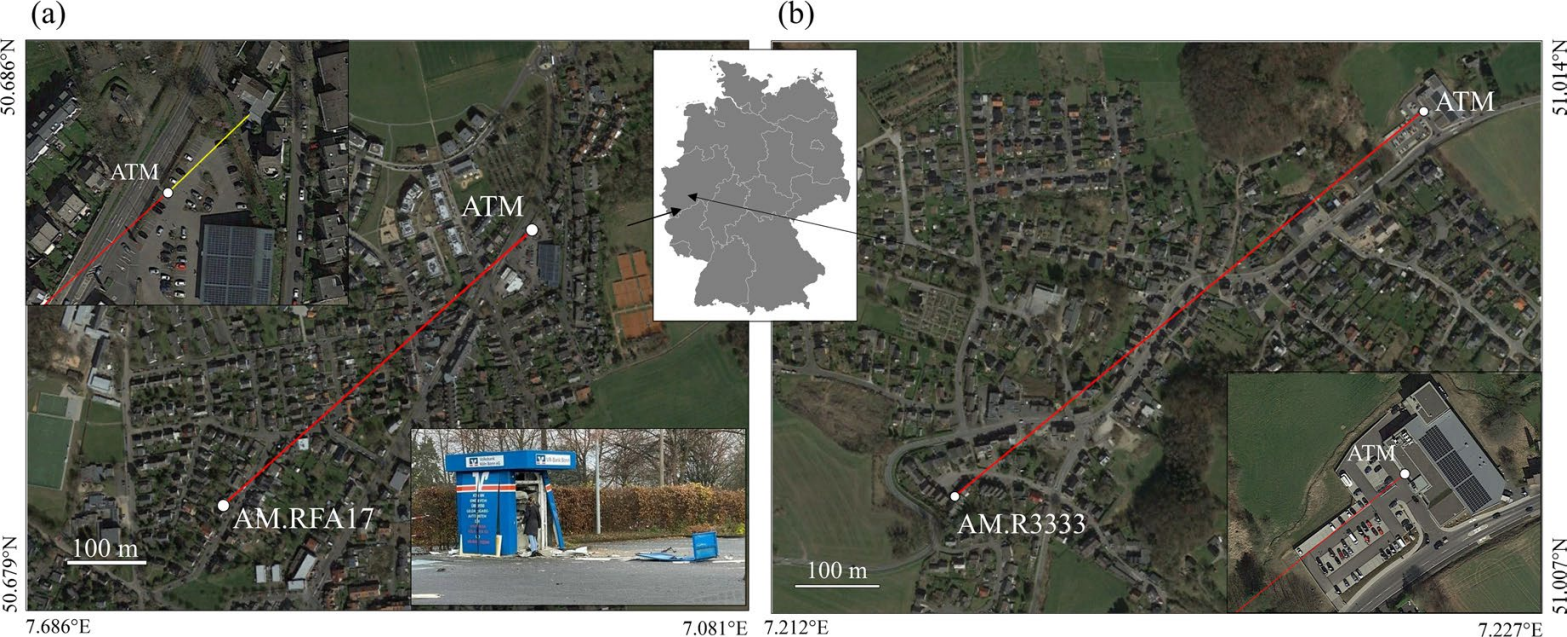
**a** Location of the Raspberry Shake citizen station AM.RFA17 in Bonn-Röttgen and the site of the ATM which was damaged on Dec. 4, 2021. The red line indicates a distance of 580 m at a bearing of 228°. The photo in the lower right corner shows the ATM after the explosions. The insert in the upper left shows the surrounding of the ATM; the yellow line measures the 50 m to the next building along projection of the bearing to the station. **b** Location of the Raspberry Shake citizen station AM.R3333 and the site of an ATM which was robbed on 23 February 2022. The red line indicates a distance of 830 m at a bearing of 320°. The insert in the lower right shows the surrounding of the ATM. The location of the two maps from (**a**) and (**b**) is indicated by arrows pointing on a map of Germany. Maps based on GoogleEarth. (Photo of ATM courtesy of A. Vogel, see “Data availability”)

On December 4, 2021, unknown suspects attempted to burglarize an ATM in Bonn-Röttgen (Fig. 3a). This ATM is not located within a building but stands alone in the parking lot of a supermarket. At 03:53 local time, the sound of a blast woke residents in the surrounding area. Macroseismic reports reached us from as far as 4-km distance from the source. Most reported that they heard two distinct booms, and it was speculated that the second might have been an echo. One of the authors (H.K.) was awakened by the explosion along with his dog Amica, who started barking (she often barks). His house is located 580 m from the ATM (Fig. 3a). On the ground floor of the house, a RS seismic station (AM.RFA17) which has been active since 2018 recorded the blasts on its velocity channel (SHZ, 50 samples per s). (The channel abbreviations are listed in the SEED Reference Manual http://www.fdsn.org/pdf/SEEDManual_V2.4.pdf, last accessed 02.2022.)

While working on the burglary incident in Röttgen, a second such burglary occurred in Kürten-Dürscheid on 23.02.2022 at 03:00 local time. Coincidentally another Raspberry Shake station (AM.R3333) was installed at a distance of 830 m on the floor of a garage of a single-family home (Fig. 3b) of one of the authors (C.F., who did not wake up), which again recorded two distinct blasts. This citizen seismological station is equipped with a seismic velocity channel (EHZ, 100 samples per s) and an air pressure channel (HDF). The ATM was located at the entrance of a supermarket building, and following newspaper reports (see data and resources), several people in the neighborhood woke up; one witness even took a video of the scene after the blasts had alarmed him. We present the data of these two robberies with four explosions, look at the nature of the second signal, and make a rough estimate of the charge weights. In the following we will use ROE and DUR to refer to the incidents in Bonn-Röttgen and Kürten-Dürscheid, respectively.

## The burglargrams

In the ROE case, the instrument was a RS1D with 50 samples per second and in the DUR case a RS&BOOM with 100 samples per second. The velocity channels of the RS instruments are equipped with a 4.5-Hz geophone with electronic extension to lower frequencies. The infrasound channel uses a MEMS temperature compensated differential pressure transducer with a bandwidth from 1 to 44 Hz. The digitizer is a 24-bit sigma-delta type with 144-dB dynamic range; for all recording parameters including poles and zeros of the transfer functions see the “Data availability” section.

**Fig. 4:**
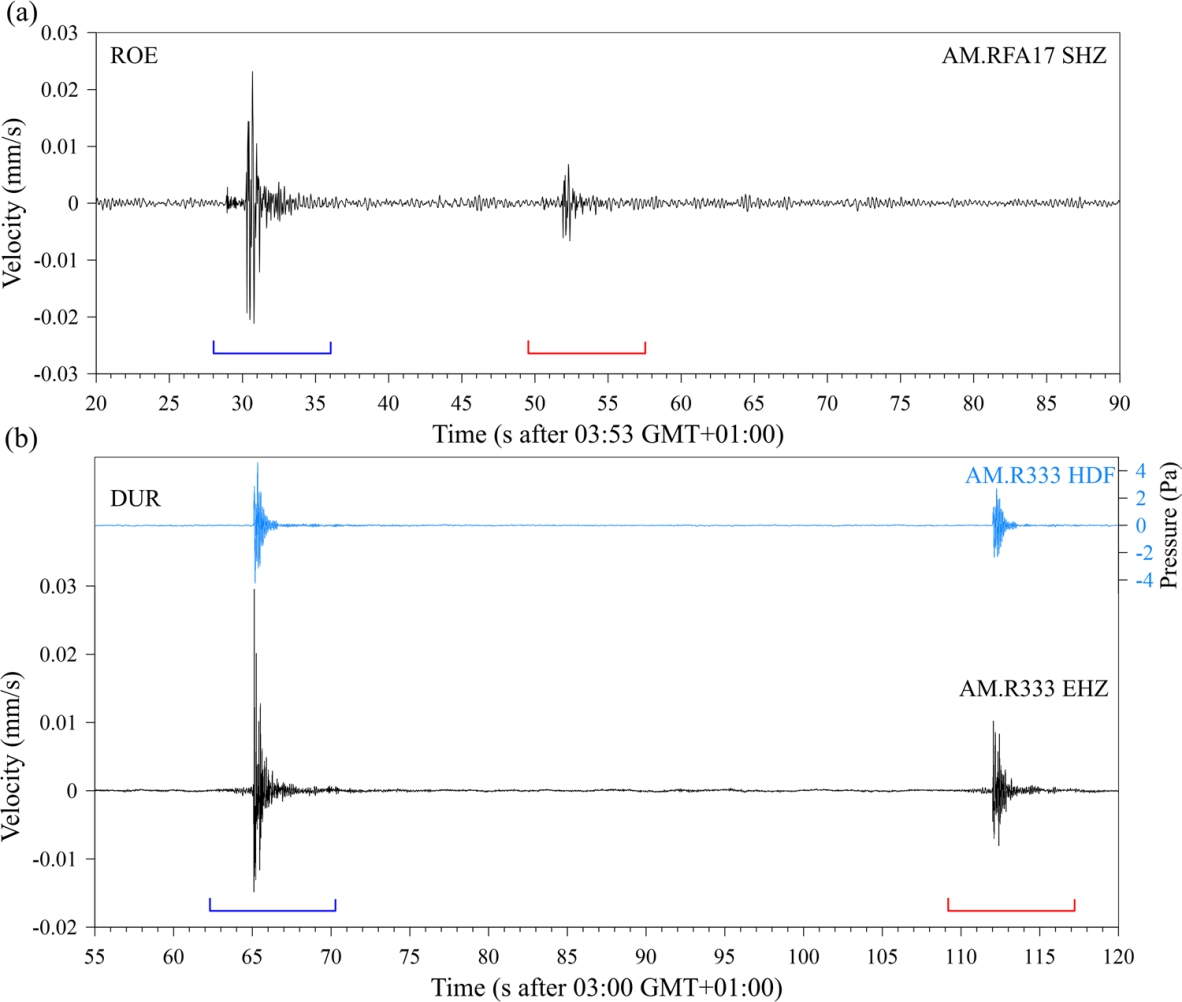
**a** Record of the vertical component of the ground velocity at RS station AM.RFA17 in a time window of 35 s starting on 2021–12-04 02:53 UTC. **b** Record of the vertical component of the ground velocity at RS station AM.R3333 in a time window of 35 s starting on 2022–02-04 02:00 UTC. The blue signal is the pressure recorded with the HDF channel of the station. The blue and red brackets in (**a**) and (**b**) indicate time windows which are shown enlarged in Fig. 5

**Fig. 5:**
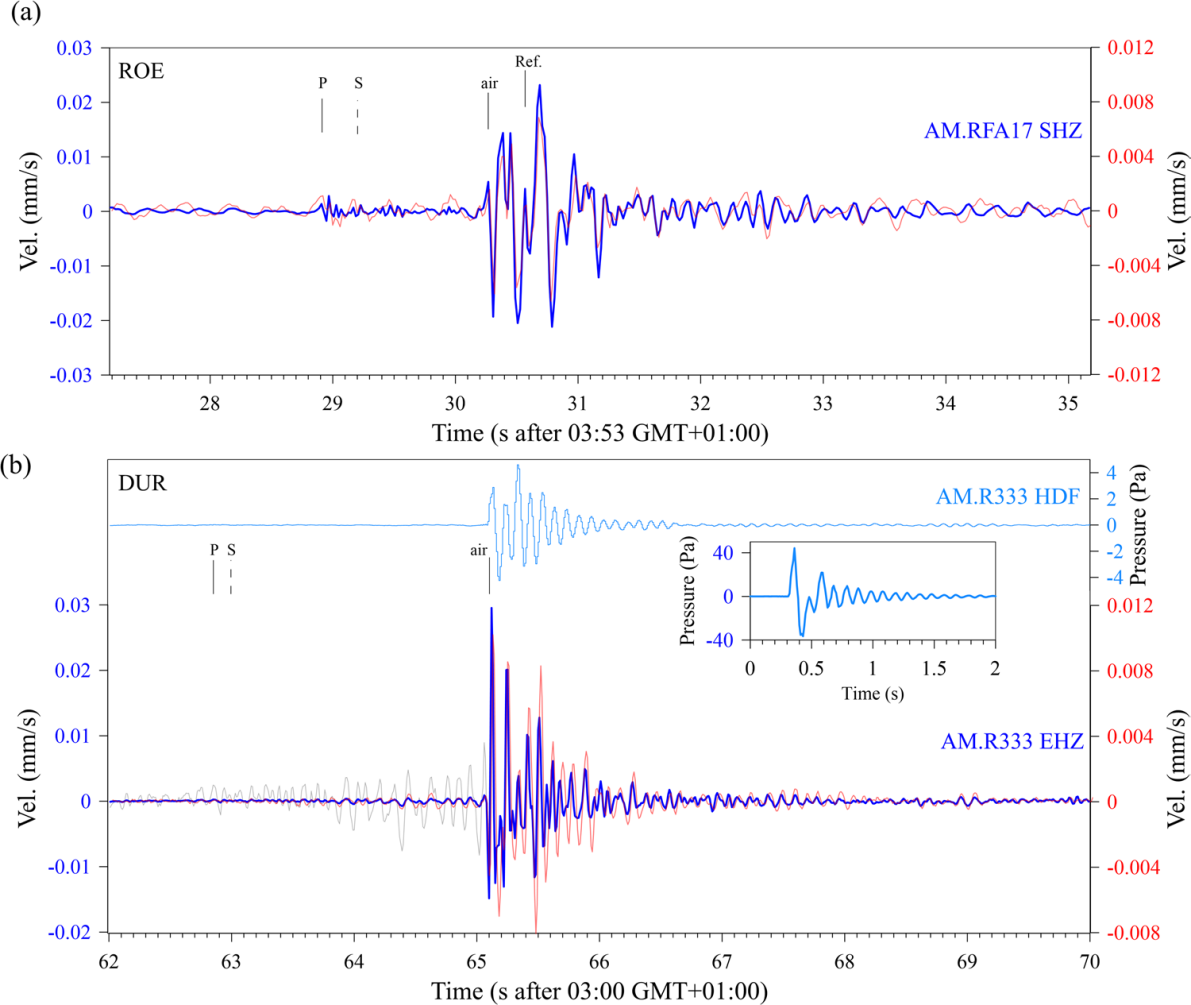
Detailed view of the seismograms of the blast events in Röttgen (**a**) and Dürscheid (**b**). The blue and red traces are in both cases the velocity seismograms of the first and second events, respectively, in time windows indicated in Fig. 4. The light blue trace in (**b**) is the pressure record of the HDF channel of the first event in Dürscheid. The insert with the blue trace is the response to a manual pressure pulse on the garage door. The gray line shows the ground velocity of the first explosion before the arrival of the airblast with a tenfold increased amplitude scale. P, S, and air indicate the estimated arrivals of the P-wave, S-wave, and the airblast, respectively. A supposed arrival of a reflection of the airblast is labeled with Ref. Station and channel IDs are given on the right end of the traces

Figure 4a shows the velocity seismogram of the RS station AM.RFA17 for the ROE case. Two events are clearly detectable with a time delay of 21.6 s between first arrivals. The peak particle velocity (PPV) of the first and second events is 0.023 and 0.007 mm/s, respectively. A detailed view of the two events is given in Fig. 5a. Comparison of the waveforms of the first and second event in Fig. 5a shows an almost perfect match, with an amplitude ratio of 3.3:1. This match indicates that the second event was not an echo. The additional time of 21.6 s would require a reflection at a distance of roughly 3.5 km. Along this travel distance and as a result of a reflection, the signal shape would have altered. The match clearly indicates a second explosion at the same location caused by a significantly smaller charge. While the arrival of the P-wave is clear for the stronger first event, it is somewhat obscured by noise for the second one; however, correlation of the two events confirms the time delay. In the seismograms in Fig. 5a, the arrivals of the P-phase and of the air blast are indicated; the S-arrival is based on the following estimate of P-, S, and sound velocities.

A weather station in the garden of the house with the citizen station RS AM.RFA17 recorded at the time of the explosion a temperature of 2.8 °C, a wind speed of 0.7 m/s at wind direction of 162°, and a relative humidity of 92%. With these values, the sound speed in air can be estimated to 333.5 m/s (http://www.sengpielaudio.com/calculator-airpressure.htm, last accessed 02.2022). The difference between the azimuth of the travel path from the source to the station (228°) and the wind direction (162°) is 66°. Thus, there is only a minor influence from wind speed of 0.28 m/s to the speed of sound with an estimate of 333.8 m/s. A time delay between the P-wave arrival and the onset of the airblast signal of 1.35 s results in the P-wave velocity, *v*_P_, of 1508 m/s, which seems reasonable for the Pleistocene clayey silt which, following the geologic map (see “Data availability”), forms the subsurface. With the assumption of a *v*_P_/*v*_S_ ratio of 1.73, the S-wave velocity, *v*_S_, is 870 m/s. However, as shown in Fig. 5a, no S-arrival can be detected on the record of the vertical component of ground motion. An absence of S-wave energy indicates that only a small fraction of the explosive’s energy was converted to seismic ground motions. In Fig. 5a, a further arrival within the airblast signal is indicated.

Figure 4b shows the velocity seismogram of the RS station AM.R3333 for the DUR case and the additional pressure record of the HDF channel. Again, two events are clearly detectable with a time delay of 49.2 s between first arrivals. The PPV of the first and second events is 0.030 and 0.012 mm/s, respectively; the peak amplitude of the pressure record is 4.6 Pa and 2.72 Pa for the first and second events, respectively. A detailed view of the two events is given in Fig. 5b. Comparison of the waveforms of the first and second event in Fig. 5a shows a good match, with an amplitude ratio of 2.5:1. At a private weather station at 9-km distance, the temperature at the time of the blasts was 4.2 °C and no wind; thus, the air wave velocity was estimated to 333.9 m/s. A very weak P-wave arrival, smaller than in the ROE case, is visible 2.25 s prior to the arrival of the airblast. This delay combined with the 830-m distance between source and receiver indicates an average P-wave velocity of 3570 m/s which is plausible for the compacted Devonian limestone which builds the subsurface. No S-wave arrival is detected in the vertical component.

The pressure signal in Fig. 5b shows a decaying reverberation after the initial onset. This reverberation has a frequency of 10.8 Hz. We made a simple test by pushing manually on the center of the garage door. The resulting pressure signal shown by the insert in Fig. 5b has a frequency of 9.8 Hz.

According to a newspaper report, the entire roof of the supermarket was temporarily uplifted by the explosion (https://www.ksta.de/region/rhein-berg-oberberg/anwohner-filmt-ueberfall-drei-maenner-sprengen-geldautomat-in-duerscheider-supermarkt-39485200?cb=1645885530345&, last accessed 02.2022).

**Fig. 6:**
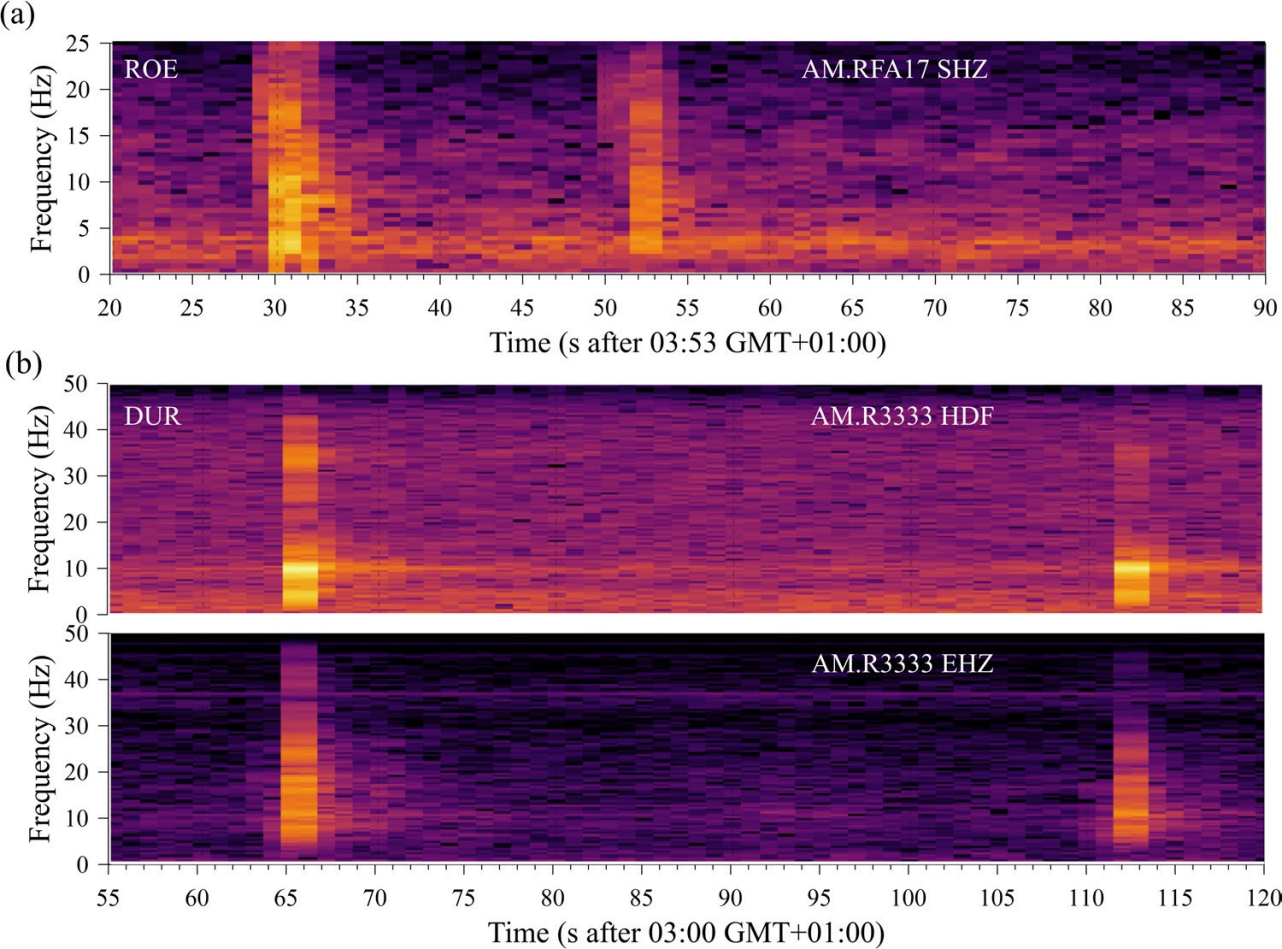
Spectrogram of the same time windows as in Fig. 4 of the velocity record of RS station AM.RFA17 in (**a**) and the pressure and velocity channel of station AM.R3333 in (**b**) (https://community.raspberryshake.org/, last accessed 01.2022)

**Fig. 7:**
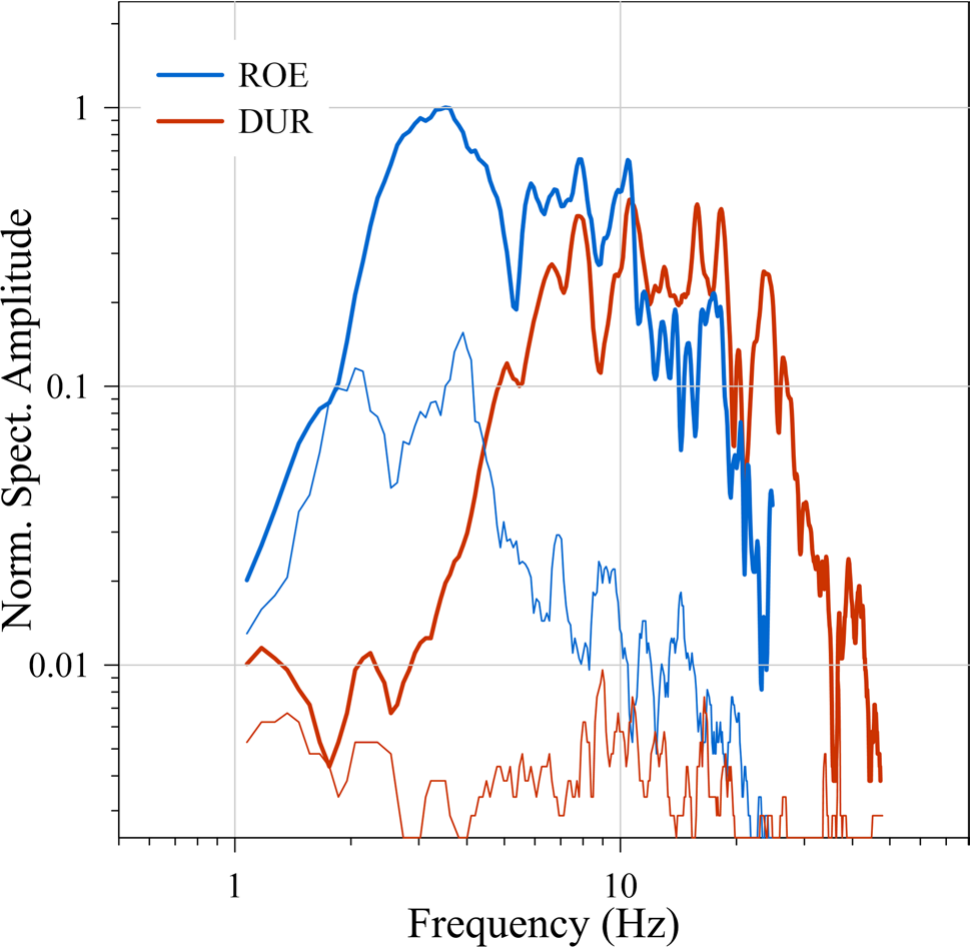
Normalized Fourier amplitude spectra of an 8-s time window around the first event of the ROE and DUR records of the vertical component of the ground velocity. The thin lines of respective color show the spectra of 8-s noise samples before the first arrivals

The spectrograms, calculated with 2-s time windows, 50% overlap, and a Hanning taper of 5%, in Fig. 6 show that in the ROE and DUR cases both events have energy in the complete frequency range covered by the RS instruments up to 25 Hz and 50 Hz in the ROE and DUR cases, respectively. The Fourier amplitude spectra of the first stronger blast events in both cases are shown in Fig. 7. Spectra were calculated from time windows of 8 s with zero padding up to 1024 samples, Hanning taper of 10% of window length and smoothed by a five-sample moving average. While the spectra roughly agree concerning the high-frequency spectral roll off, there is much more signal energy at low frequencies below 5 Hz in the ROE case than at DUR.

## Estimate of the charge weight

A well-constrained estimate of the charge weight of the ROE explosions is hampered by the fact that the station AM.RFA17 is only equipped with a single (vertical) component sensor, and the type of explosives is unknown. Further, the station is located on the ground floor of a single-family home, thus possibly shielding part of the air wave overpressure from the seismic record. In the DUR case, also only a vertical ground motion signal is available; however, the data from the pressure sensor can be used in addition. Here the shielding should be somewhat different than in the ROE case, as the large metal garage door probably has smaller effects than the massive building with insulating windows in the ROE case.

As the P-wave seismic signals are very weak in both cases, we attempted to use the PPV of the air wave signal to set limits to the size of the charge. Several previous studies have estimated the acoustic-to-seismic ground coupling coefficients for the ground velocity (VCC) (e.g., McDonald and Goforth 1969; Bass et al. 1980; Sabatier et al. 1986; Edwards et al. 2007; Matoza & Fee 2014; Novoselov et al. 2020). The empirically estimated coefficients that translate the overpressure to a PPV of the ground include a wide range between 0.4 and 14 µms^−1^ Pa^−1^, depending on the frequency of the pressure wave, distance from source, and the subsurface conditions. The sources for the overpressure in these studies range from sonic booms and volcanic events to explosives. Novoselov et al. (2020) performed a series of controlled experiments with explosives and firework-rockets and found values for VCC between 1.99 and 2.74 µms^−1^ Pa^−1^ at frequencies between 48 and 331 Hz at distances from 0 to 30 m. These frequencies are higher than the observed 6–10 Hz in case of the ROE and DUR explosions. However, Matoza and Fee (2014) report VCC in the same range as Novoselov et al. (2020) for frequencies between 7 and 12 Hz for sonic booms from the Mount St. Helens explosion in 2005.

If we take the peak pressure value from the DUR record combined with the PPV, the resulting VCC is 6.42 µms^−1^ Pa^−1^. This value will be assumed for the VCC and additionally for comparison to the one of 2.4 ms^−1^ Pa^−1^, which is in the middle of the range determined by Novoselov et al. (2020) and Matoza and Fee (2014).

The ICI Handbook of Blasting Tables (ICI 1971) suggests a basic air vibration attenuation equation:

1 $$P=A{\left(\frac{D}{\sqrt[3]{W}}\right)}^{a}$$

where *P* is the pressure in kPa; *A* and *a* are a site constant and exponent, respectively; *D* is the distance in m; and *W* the TNT equivalent charge weight in kg. For unconfined surface charges, based on empirical data, the handbook suggests the equation:

2 $$P=185{\left(\frac{D}{\sqrt[3]{W}}\right)}^{-1.2}$$

**Fig. 8:**
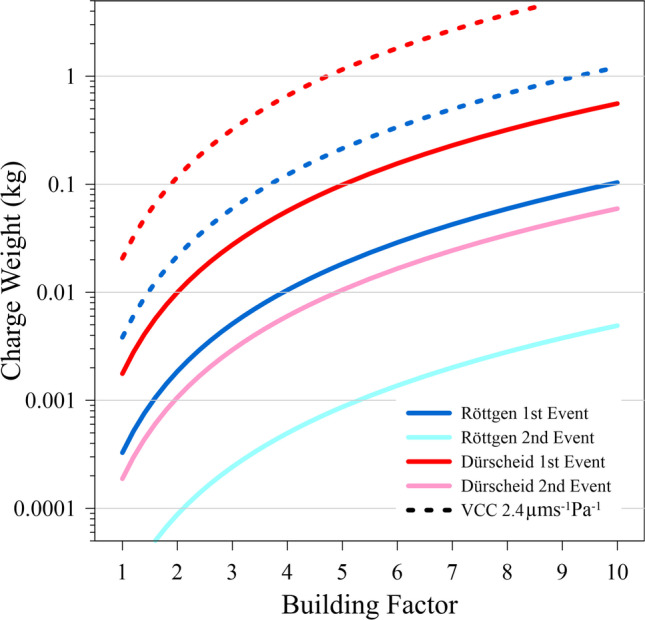
Charge weight of the ROE and DUR ATM burglary explosions estimated with Eq. (2) for assumed shielding of the air wave by the building with factors ranging from 1 to 10. The data are shown for the first and the second event (see Fig. 4) as indicated by the legend. The solid curves are the values for a VCC of 6.42 µms^−1^ Pa^−1^. In addition, the dashed lines show the results for the first blasts in both cases if a VCC of 2.4 µms^−1^ Pa.^−1^ is assumed

Using the abovementioned range of VCC values and Eq. (2), the charge weight can be estimated based on the PPV for the first and second event in both cases. As the measuring points were both located in a building, we further assumed building attenuation factors between 1 and 10. The results are shown in Fig. 8.

## Discussion and conclusion

In the attempt to rob an ATM in Bonn-Röttgen, Germany, and the completed robbery of another ATM in Kürten-Dürscheid, Germany (Fig. 3), the burglars used explosions to demolish the casing of the machines. In both cases, citizen seismic stations recorded ground motions of these attacks with a Raspberry Shake station at a distance of 580 m and 830 m, respectively. The records (Figs. 4 and 5) show the two explosion events separated by a time delay of 21.6 s and 49.2 s. The main measured and estimated parameters for both cases are summarized in Table 1. The excellent match of the wave forms of the first and second event in the ROE case contradicts the assumption of some auditory observers that the second blast was an echo effect. The same can be concluded for the DUR robbery.

**Table 1:** Main measured and estimated parameters of the seismograms from the Röttgen (ROT) and Dürscheid (DUR) ATM burglaries

	ROE	DUR
Date	2021–12-04	2022–02-23
Local Time 1. Event	03:53:29	03:01:03
Delay 1. to 2. Event	21.6 s	49.2 s
Station ID	AM.RFA17	AM.R3333
Distance	580 m	830 m
Azimuth	228°	320°
*v*_air_	333.5 m/s	333.9 m/s
*v*_p_	1508 m/s	3570 m/s
PPV 1. event	0.023 mm/s	0.030 mm/s
PPV 2. event	0.007 mm/s	0.012 mm/s
Meas. Peak Pressure 1. Event	-	4.60 Pa
Meas. Peak Pressure 2. Event	-	2.72 Pa
Est. Peak Pressure 1. Event	3.62 Pa	4.60 Pa
Est. Peak Pressure 2. Event	1.07 Pa	1.88 Pa
Ratio Charge 1./2. Event	21:1	9.4:1

There are later arrivals in the recorded ground motion signals induced by the airblasts. Gitterman and Hofsttetter (2014) have developed empirical relations between the yield of an explosion and the so-called secondary shock effect which is formed by successive implosion of rarefaction waves from the contact surface between explosion products and the air. Budakoğlu (2022) for example has used the time delays of the secondary shock effect to estimate the charge weight of accidental fireworks explosions in Turkey. However, in this analysis the secondary arrivals are too late to be interpreted as secondary shock effects. The arrival indicated in Fig. 5a in the ROE case at 0.3 s after the first arrival agrees well with the reflection time for the pressure wave from the closest building to the ATM at a distance of 50 m.

The lower frequencies of the ground motion in the ROE case compared to the DUR case, which are evident in the Fourier spectra (Fig. 7), are probably an effect of the different subsurface structure. The Pleistocene clayey silt at ROE with a P-wave velocity of 1508 m/s is softer than the Devonian compacted limestone at DUR with 3570 m/s and therefore results in a stronger response at lower frequencies to the impacting airblast.

The similar signal shape and frequency of the airblast signal from the first explosion in the DUR case and the reaction to a manual push on the garage door (Fig. 5) where the instrument is installed indicates that the reverberating character is a local site effect.

Due to the lack of definite knowledge of parameters, the estimate of the TNT equivalent charge weight for the explosions contains large uncertainties. On one hand, the estimate of the relation between the air pressure and the ground velocity is arguable. In the DUR case, based on the pressure measurement, the VCC estimate of 6.42 µms^−1^ Pa^−1^ is well in the range of published values from 0.4 and 14 µms^−1^ Pa^−1^. However, the ATM demolishing was not a controlled experiment and the station location in a garage with a closed door can have significant influence on the VCC. Despite this, based on this VCC and (arbitrarily) assuming a shielding factor in the DUR case of 4, the charge weight for the first event comes to 57 g of TNT equivalent (Fig. 8). The strong dependence on the VCC is evident, as under the same conditions with a VCC of 2.4 µms^−1^ Pa^−1^ the charge weight is predicted to be 670 g. The shielding of the residential building in the ROE case is assumed to be significantly larger than that of the garage in the DUR case. If charge weights for the first explosions would have been the same in both cases, this would indicate a shielding factor of about 8 in the ROE case. Without proper calibration, both effects cannot be further constrained and thus the uncertainties of the absolute charge weights remain. A reduction of the uncertainties would require controlled in situ tests, which would not be permitted by the authorities.

Interesting and much more certain are the ratios of the charges used for the first and second explosions in both cases, which are 21:1 and 9.4:1 for ROE and DUR, respectively. The first blasts were probably used to open the casing of the ATMs and to get access to the cassettes containing the cash. The following blasts then were probably applied to open the cassettes, which, following the police reports, did not work in the ROE case, but was “successful” in the DUR case.

Our expertise is only to interpret the seismic records, but in such a forensic analysis, a few further thoughts are reasonable. There are striking similarities between the two cases, (1) attack of an ATM at a supermarket at the periphery of a residential area; (2) both attacks were between 3 and 4 AM local time; (3) two explosions were used; (4) the time interval between the two blasts is short, less than a minute. The latter indicates that the charges for the blasts must have been well prepared in advance; otherwise, it would have taken longer than 20 to 50 s to implement and detonate the second one. If we speculate that the attacks were made by the same suspects, the significantly increased charge of the second blast in the DUR case, compared to the ROE case, might be a reaction to the failure to open the cassette in the ROE case.

Both incidents advocate for the use of citizen seismographs like the Raspberry Shake instrument that can produce interesting and useful data from non-earthquake events. The amount of information would have been even better if three components of ground motion plus an air pressure channel have been available at both sites. The study also shows that wherever possible sensors should be installed in the free field, particularly those with an infrasound detector. Our observations will probably not help to solve the burglary case; however, they might be a motivation for the installation of more citizen seismographs (e.g., at ATM sites).

## Data Availability

The seismic data of the RS stations is freely available at https://dataview.raspberryshake.org; the same resource was used to calculate the spectrograms and all instrument specifications can be found at https://manual.raspberryshake.org/metadata.html. Maps of the study area are based on Google Earth Pro. Data source for the crime statistics is https://de.statista.com/statistik/daten/studie/585381/umfrage/anzahl-der-diebstaehle-durch-sprengung-eines-geldautomaten-in-deutschland/ based on data from the Federal Criminal Police Office (https://www.bka.de/EN/Home/home_node.htmlhttps://de.statista.com/statistik/daten/studie/ 585,381/umfrage/anzahl-der-diebstaehle-durch-sprengung-eines-geldautomaten-in-deutschland/). Speed of sound was estimated with http://www.sengpielaudio.com/calculator-airpressure.htm. Acoustic pressure and sound levels were converted with http://www.sengpielaudio.com/Rechner-schallpegel.htm. The geologic amp for the Röttgen area can be reached at https://www.opengeodata.nrw.de/produkte/geologie/geologie/ GK/ISGK25/GK25analog/. The newspaper report on the Dürscheid explosion was retrieved at https://www.ksta.de/region/rhein-berg-oberberg/anwohner-filmt-ueberfall-drei-maenner-sprengen-geldautomat-in-duerscheider-supermarkt-39485200?cb=1645885530345&. The photo of the damaged ATM was provided by A. Vogel (axel.vogel@t-online.de). All online-sources were last accessed in February 2022.
